# Comparison of the Effect of Three Irrigation Techniques and Root Canal Preparation Size on Sodium Hypochlorite Penetration into Root Canal Dentinal Tubules

**DOI:** 10.1155/2021/6612588

**Published:** 2021-03-31

**Authors:** Pakit Tungsawat, Pondpun Arunrukthavorn, Phawinee Phuntusuntorn, Suphakit Opatragoon, Pharsiri Sirirangsee, Surat Inklub

**Affiliations:** Department of Endodontics, College of Dental Medicine, Rangsit University, Pathum Thani, Thailand

## Abstract

**Objective:**

This study aims to compare the effects of conventional syringe irrigation (CSI), passive ultrasonic irrigation (PUI), and innovative sonic-powered irrigation (EDDY) on the penetration of sodium hypochlorite (NaOCl) solutions into root canal dentinal tubules at different levels of the root canal.

**Materials and Methods:**

One hundred ninety-two extracted first mandibular premolars of 17- to 25-year-old patients were decoronated 13 mm from the root apices and separated into two groups according to apical preparation sizes (APS) 25 and 40. The root canals were dried with a paper point and stained in crystal violet for 72 hours. Each APS group was separated into three groups according to irrigation techniques, as follows: CSI, PUI, and EDDY. Roots were perpendicularly resected to the long axis at three levels (coronal, middle, and apical). Photomicrographs were taken of all three cross-sections of each tooth under a stereomicroscope. The depth of the bleached zone was measured with ImageJ software. The data were analyzed by Welch's analysis of variance and an independent *t*-test (*p* = 0.05).

**Results:**

No penetration was found at the apical level in the CSI25, CSI40, and PUI25 groups. The EDDY25 and EDDY40 groups showed the most significant penetration at the middle and apical levels compared to the other groups (*p* < 0.05).

**Conclusions:**

Within the limitations of this study, irrigation techniques and APS affect the penetration depth of NaOCl into the root canal dentinal tubules. In terms of irrigation techniques, the penetration was deepest when EDDY was used, followed by PUI and CSI. In terms of APS, NaOCl penetrated deeper into APS40 than APS25. The use of the EDDY irrigation technique in APS25 can improve the penetration of NaOCl into root canal dentinal tubules at the apical level.

## 1. Introduction

The success of root canal treatment depends on the elimination of intraradicular infection [1, 2]. Bacteria and endotoxins in the root canal can penetrate approximately 300–500 micrometers (*μ*m) into dentinal tubules [3, 4]. Root canal preparation, irrigation, and medication are core methods to eradicate bacteria from the dentinal tubule and root canal system [5]. Attempts have been made to improve the efficiency of root canal irrigation and medication. A recent study reveals the propolis is superior to calcium hydroxide in the antimicrobial properties and the prevention of postoperative pain [6–8]. In terms of root canal irrigation, various devices have been developed to enhance the efficiency of the root canal irrigants. The apical region of the root canal is the most difficult section to clean due to the complexity of the anatomy [9]. The cleanliness of the apical third is determined by apical preparation size (APS) and irrigation protocol. APS affects the insertion distance of the needle tip, the dynamics of irrigation flow, and the disinfecting effect of irrigation [10]. An increase in the APS significantly improved root canal disinfection [11]. Histological analysis showed that a larger APS was cleaner than a smaller size when the root canals were irrigated with a syringe and needle. When irrigation was performed with passive ultrasonic techniques, the small prepared canals were as clean as the largely prepared canals [12].

Sodium hypochlorite solution (NaOCl) is commonly used as a root canal irrigant because of its antimicrobial activity and tissue dissolution ability [13]. A previous study showed that a 6% NaOCl solution could penetrate 300 *μ*m into dentinal tubules at 45°C with 20 minutes of exposure [14]. The irrigation techniques could affect the penetration efficacy of NaOCl. Various root canal irrigation techniques have been used in endodontic treatment, conventional syringe irrigation (CSI). Passive ultrasonic irrigation (PUI) is based on the use of an ultrasonic instrument to activate the irrigant in the root canal. Innovative sonic-powered irrigation (EDDY) uses flexible polyamide tips to prevent cutting dentin and changing root canal morphology during sonic activation at high frequency, which is useful in removing debris and organic tissues from canal walls [15, 16]. A previous study showed that PUI and EDDY were similar in activating the irrigant solution to penetrate the dentinal tubules with a large APS [17]. However, this study compares the effect of different activation techniques together with different APSs.

This study aimed to compare the effect of three different irrigation techniques and two different APSs on the penetration depth of NaOCl into the root canal dentinal tubules.

## 2. Materials and Methods

One hundred ninety-two permanent mandibular first premolar teeth that were extracted from 17- to 25-year-old patients during orthodontic treatment with a single root canal and no previous root canal treatment were used in this study. Radiographs of the teeth were taken in both the mesiodistal and buccolingual aspects to confirm a single root canal. Teeth with root lengths shorter than 13 mm, restoration, caries, cracks, fractures, or immature apexes were excluded. The samples were stored in 0.1% thymol immediately after extraction until use.

The teeth were decoronated by a carborundum disc 13 mm from root apices. A size 10 stainless steel k-file (VDW GmbH, Munich, Germany) was used to achieve apical patency, and a size 15 stainless steel k-file (VDW GmbH, Munich, Germany) established a working length 1 mm shorter from the apical foramen. If a size 15 stainless steel k-file was loose at the apical foramen, the root was excluded. The samples were divided into two groups according to APS (*n* = 192). Each tooth was instrumented with Mtwo rotary files (VDW GmbH, Munich, Germany). Group 1 (APS = 25, *n* = 96), the root canals were prepared to size 25. Group 2 (APS = 40, *n* = 96), the root canals were prepared to size 40. The instrumented canal was irrigated with 2 ml of 2.5% NaOCl solution for 30 sec. The irrigant was delivered via a 27-gauge, open-ended needle (Nipro, Ayutthaya, Thailand) by placing the tip 3 mm short of the working length after each file change. Final irrigation was performed with 5 ml of 17% ethylenediamine tetraacetic acid (EDTA) for 1 minute, and the canal was flushed with 5 ml of distilled water and dried with three pieces of paper points. The root apex was sealed with composite resin to create a closed system.

The root canals were stained by submerging in crystal violet at room temperature for 72 hours. Crystal violet was renewed every 12 hours. Then, the root canals were washed with 20 mL of distilled water. The samples were randomly divided into six experimental groups (*n* = 180) according to irrigation techniques and APS and two control groups (*n* = 12). The irrigation techniques were performed as follows:

- CSI: the irrigant was applied with a 27-gauge, open-ended needle. The needle tip was placed 3 mm shorter than the working length, and the canals were irrigated by using 6 mL of 2.5% NaOCl for 120 sec.
- PUI: the irrigant was applied with a 27-gauge, open-ended needle. The irrigation needle was inserted 3 mm shorter from the working length, and the canal was irrigated with 2 mL of 2.5% NaOCl for 20 sec. Then, the irrigant was activated with an ultrasonic file size of 20 (IRRI 20; Irri-safe^TM^, Acteon®, Merignac, France) by insertion 3 mm shorter than the working length for 30 sec, followed by 2 mL with the CSI technique for 20 sec, PUI for 30 sec, and 2 mL with CSI for 20 sec. The total volume of irrigant was 6 mL, and the total time of irrigation was 120 sec.
- EDDY: the irrigant was applied with a 27-gauge, open-ended needle. The irrigation needle was inserted to 3 mm shorter than the working length, and the canal was irrigated with 2 mL of 2.5% NaOCl for 20 sec and then activated with EDDY (VDW GmbH, Munich, Germany) by insertion to 3 mm shorter than the working length for 30 sec, followed by 2 mL with CSI for 20 sec, then EDDY for 30 sec, and followed by 2 mL with CSI for 20 sec. The total volume of irrigant was 6 mL, and the total time of irrigation was 120 sec.

A negative control group (*n* = 6): the root canals were prepared with the Mtwo rotary up to size 40/04 and then dyed with crystal violet only to evaluate the penetration of crystal violet.

A positive control group (*n* = 6): the root canals were prepared with the Mtwo rotary up to size 40/04, dyed with crystal violet, and stored with NaOCl to evaluate the bleaching effect of NaOCl for 72 hours.

**Figure 1:**
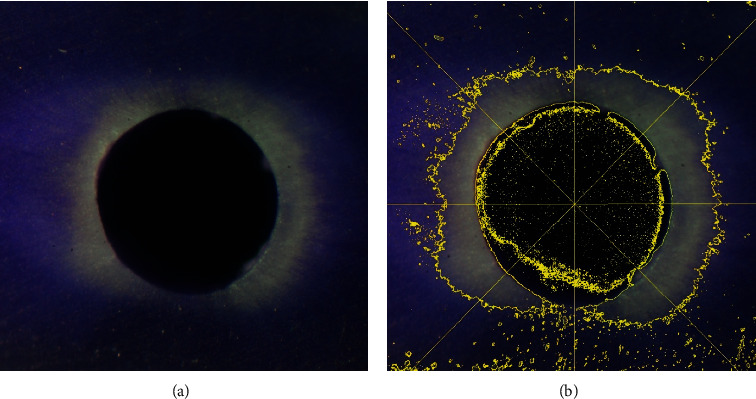
(a) A bleached crystal violet image of the EDDY40 group under a stereomicroscope (original magnification, 40x). (b) Determining the bleached depth by ImageJ software.

All specimens were irrigated with distilled water (2 mL) and dried with three pieces of paper point, then sectioned perpendicular to the long axis of the root by a carborundum disc at 3 mm from apex to represent an apical level, 6 mm from apex to represent the middle level, and 10 mm from apex to represent a coronal level. Then, the cross-sectional area of all specimens was taken as a photograph with a 16-megapixel digital camera (Olympus OM-D-E-M10) attached to the stereomicroscope (Motic SMZ-168 TP) with a magnification of 16x for the coronal section, 25x for the middle section, and 40x for the apical level (Figure 1(a)). The bleaching depth was measured with ImageJ software by setting 8 points on a virtual clock face per section at degrees 0, 45, 90, 135, 180, 225, 270, and 315 (Figure 1(b)).

The penetration of NaOCl into the dentinal wall of the root canal presented as the bleached area of crystal violet. In the positive control group, the bleached depth of crystal violet was observed to be more than four-fifths of the thickness of the root canal wall. In the negative control group, the dark purple color of crystal violet was present throughout the thickness of the root canal wall.

### 2.1. Statistical Analysis

The data were analyzed using SPSS 24.0 for Windows (SPSS Inc., Chicago, IL). The Kolmogorov–Smirnov test and Levene's test were used to test the normality and homogeneity of variants of each experimental group, respectively. The penetration depth was normally distributed. However, homogeneity of variance was not achieved, so differences in the penetration depth between each irrigation technique and root level were compared by Welch's ANOVA, and then, multiple comparisons were performed by Dunnett's T3. The significance level was set at 0.05.

## 3. Results

The penetration depth of each group is shown in Table 1. The highest penetration depth was at the coronal level of the CSI40 group. No penetration was observed at the apical level in the CSI25, CSI40, and PUI25 groups.

At the coronal level, the penetration depth was not different between the APS of each irrigation technique. At the middle and apical levels, there was a statistically significant difference in penetration depth between APSs, as size 40 penetrated deeper than size 25 in all irrigation techniques (*p* < 0.05), except at the apical level of the CSI group.

EDDY showed significantly deeper penetration than PUI and CSI at the middle and apical levels in both APSs (*p* < 0.05). However, there was no difference in the penetration depth between the middle and apical levels in the EDDY25 group and between the coronal and middle levels in the EDDY40 group.

## 4. Discussion

The reduction of bacteria in the root canal system to levels that are compatible with periapical tissue healing significantly affects the outcome of root canal treatment [18]. NaOCl is the main disinfectant solution used in root canal treatment. This study aimed to compare the efficiency of CSI, PUI, and EDDY and different APS on the penetration of NaOCl solutions into root canal dentinal tubules at different levels of the root canal. The penetration was determined by the bleached area of crystal violet in the dentinal tubules.

The CSI group had the deepest penetration depth at the coronal level, followed by PUI and EDDY. The teeth in this study were decoronated. The PUI and EDDY techniques used a vibration tip that spreads out the irrigant from the coronal level of the canal; therefore, the contact time between the irrigant and root canal surface at the coronal level in the PUI and EDDY groups was less than that in the CSI group, which did not spread out of the irrigant. However, at the middle and apical levels, there was no loss of irrigant in the canal throughout the period of activation; therefore, the results of PUI and EDDY were more effective than CSI, in agreement with a previous study [15].

In terms of the root canal level, the coronal level of the root canal had the greatest penetration depth of NaOCl in all groups because the coronal level had the largest diameter and highest density of dentinal tubules. The diameters of the dentinal tubules were 4.32 ± 1.00, 3.75 ± 1.48, and 1.73 ± 0.93 *μ*m in the coronal, middle, and apical portions of the root, respectively [19, 20]. The penetration at the coronal level between APS25 and APS40 for each irrigation technique was no different because of the similar size of the root canal at the coronal level in both groups. The different apical preparation sizes did not alter the original shape at the coronal level of the root canal because the diameter at the coronal level of the root canal of the premolar is significantly larger than that at the middle and apical levels [21].

No penetration was found in the apical portion of the CSI25, CSI40, and PUI25 groups. This could be because the position of the needle and activator tips in this study was 3 mm short of the working length. In contrast with other studies, this study used 27G needles with an outer diameter of 0.41 mm, and the smallest size of the prepared root canal was 25.06 tapers. The diameter of the prepared root canal was 25.06 at 2 and 3 mm short working lengths of 0.37 mm and 0.43 mm, respectively. Then, the needle tip could be freely inserted deepest at 3 mm from the working length. Because of the limitation of the needle tip and canal preparation size, the positions of the needle tip and activator tips were set at a working length of 3 mm in all groups. In addition, the flowability of the irrigant was limited at the apical level of the small preparation size root canal [22–24]. Further reason could be caused by the apical vapor lock effect. The apical vapor lock effect is entrapment of the air bubble during irrigation in the close-ended channel and occurs in the apical portion of the root canal and interrupts the penetration of irrigants [25]. Previous studies have shown that CSI cannot eradicate air bubbles, but sonic activation can be considered an effective method for vapor lock reduction [26, 27]. A previous study found that the small APS impeded the penetration of irrigants at the apical level due to diminished contact between irrigants and canal walls [28]. The insertion depth of the syringe needle irrigation depends on the size and morphology of the canal [29]. The large APS enhances the flow of irrigants at the apical level [30].

The results of this study showed that EDDY was more effective than PUI at the middle and apical levels, whereas PUI was superior to EDDY at the coronal level. In contrast, in a previous study, the penetration depth of NaOCl for the whole canal of the EDDY and PUI techniques for the APS 40.06 taper was not different [17]. The inconsistent results could be caused by the difference in the sample size between the two studies. In this study, the sample size of each group was 30, whereas another study used 15 samples in each group. As a result of a sufficiently large sample size, the data of each group were normally distributed, and a statistically significant difference between the groups could be detected.

At the apical level of APS 25, EDDY could activate NaOCl to penetrate dentinal tubules, while PUI and CSI were ineffective. The EDDY tip had a diameter of 0.2 mm that was activated in 3-dimensional movement. According to the manufacturer, it works at a high frequency of approximately 6000 Hz and an amplitude of 160 *μ*m, while the ultrasonic tip has a frequency of 28–32 kHz and amplitude of 28 *μ*m. Therefore, in the root canal, the amplitude of the oscillation may have more effect on the penetration of the irrigant than the oscillating frequency. Another advantage of the EDDY tip is that it is made from flexible polyamide that prevents cutting the root canal dentine wall [31].

Each irrigation technique has different characteristics of fluid dynamics. A previous study on the irrigation dynamics of different irrigation techniques found that PUI has maximum velocity and high intensity of turbulence flow at the apical portion [32]. PUI has more maximum wall shear stress than CSI, which supports the result of this study that PUI has more penetration depth of NaOCl than CSI when the canal was APS 40. However, the canal size of APS 25 could limit the effectiveness of the ultrasonic tip to generate acoustic streaming and cavitation because the tip may contact the root canal wall during activation [33].

## 5. Conclusion

Within the limitations of this study, irrigation techniques and APS affect the penetration depth of NaOCl into root canal dentinal tubules. In terms of irrigation techniques, the penetration was deepest when EDDY was used, followed by PUI and CSI. In terms of APS, NaOCl penetrated deeper into APS40 than APS25. The use of the EDDY irrigation technique in APS25 can improve the penetration of NaOCl into the dentinal tubules at the apical level.

## Figures and Tables

**Table 1:** The penetration depth of sodium hypochlorite into the dentinal wall of the root canal. Means sharing the same superscript are not significantly different from each other (*p* ≥ 0.05).

Techniques	Preparation size	Root canal level	Penetration depth (mean ± SD)
CSI	25	Coronal	899.44 ± 95.44^a^
		Middle	26.41 ± 14.30^i^
		Apical	0
	40	Coronal	902.43 ± 67.54^a^
		Middle	229.64 ± 32.77^c,d^
		Apical	0

PUI	25	Coronal	651.40 ± 138.02^b^
		Middle	110.62 ± 11.88^g, h^
		Apical	0
	40	Coronal	685.07 ± 44.78^b^
		Middle	201.31 ± 36.24^d, e^
		Apical	109.30 ± 35.96^g, h^

EDDY	25	Coronal	318.94 ± 29.51^c^
		Middle	148.56 ± 88.96^e, f, g^
		Apical	117.47 ± 21.20^f, h^
	40	Coronal	369.12 ± 192.57^c, d^
		Middle	304.72 ± 93.43^c^
		Apical	180.87 ± 97.36^d, f^

## Data Availability

The data used to support the findings of this study are available from https://drive.google.com/file/d/1teyvRDXkWRnY3ojj44FhggXgfDwPZoVK/view?usp=sharing.

## References

[1] Schilder H. (1974). Cleaning and shaping the root canal. *Dental Clinics of North America*.

[2] Tabassum S., Khan F. R. (2016). Failure of endodontic treatment: the usual suspects. *European Journal of Dentistry*.

[3] Berkiten M., Okar I., Berkiten R. (2000). In vitro study of the penetration of Streptococcus sanguis and Prevotella intermedia strains into human dentinal tubules. *Journal of Endodontics*.

[4] Meryon S. D., Brook A. M. (1990). Penetration of dentine by three oral bacteria in vitro and their associated cytotoxicity. *International Endodontic Journal*.

[5] Byström A., Sundqvist G. (1981). Bacteriologic evaluation of the efficacy of mechanical root canal instrumentation in endodontic therapy. *European Journal of Oral Sciences*.

[6] Shrivastava R., Rai V. K., Kumar A. (2015). An in vitro comparison of endodontic medicaments propolis and calcium hydroxide alone and in combination with ciprofloxacin and moxifloxacin against Enterococcus faecalis. *The Journal of Contemporary Dental Practice*.

[7] Khurshid Z., Naseem M., Zafar M. S., Najeeb S., Zohaib S. (2017). Propolis: a natural biomaterial for dental and oral healthcare. *Journal of Dental Research, Dental Clinics, Dental Prospects*.

[8] Shabbir J., Qazi F., Farooqui W., Ahmed S., Zehra T., Khurshid Z. (2020). Effect of Chinese propolis as an intracanal medicament on post-operative endodontic pain: a double-blind randomized controlled trial. *International Journal of Environmental Research and Public Health*.

[9] Kuttler Y. (1955). Microscopic investigation of root apexes. *The Journal of the American Dental Association*.

[10] Tziafas D., Alraeesi D., Al Hormoodi R., Ataya M., Fezai H., Aga N. (2017). Preparation prerequisites for effective irrigation of apical root canal: a critical review. *Journal of Clinical and Experimental Dentistry*.

[11] Rodrigues R. C. V., Zandi H., Kristoffersen A. K. (2017). Influence of the apical preparation size and the irrigant type on bacterial reduction in root canal-treated teeth with apical periodontitis. *Journal of Endodontics*.

[12] Lee O. Y. S., Khan K., Li K. Y. (2019). Influence of apical preparation size and irrigation technique on root canal debridement: a histological analysis of round and oval root canals. *International Endodontic Journal*.

[13] Pinheiro S. L., Silva C. C. D., Silva L. A. D. (2018). Antimicrobial efficacy of 2.5% sodium hypochlorite, 2% chlorhexidine, and ozonated water as irrigants in mesiobuccal root canals with severe curvature of mandibular molars. *European Journal of Dentistry*.

[14] Zou L., Shen Y., Li W., Haapasalo M. (2010). Penetration of sodium hypochlorite into dentin. *Journal of Endodontics*.

[15] Urban K., Donnermeyer D., Schäfer E., Bürklein S. (2017). Canal cleanliness using different irrigation activation systems: a SEM evaluation. *Clinical Oral Investigations*.

[16] Plotino G., Grande N. M., Mercade M. (2019). Efficacy of sonic and ultrasonic irrigation devices in the removal of debris from canal irregularities in artificial root canals. *Journal of Applied Oral Science*.

[17] Galler K. M., Grubmüller V., Schlichting R (2019). Penetration depth of irrigants into root dentine after sonic, ultrasonic and photoacoustic activation. *International Endodontic Journal*.

[18] Siqueira J. F., Rôças I. N. (2008). Clinical implications and microbiology of bacterial persistence after treatment procedures. *Journal of Endodontics*.

[19] Carrigan P. J., Morse D. R., Furst M. L., Sinai I. H. (1984). A scanning electron microscopic evaluation of human dentinal tubules according to age and location. *Journal of Endodontics*.

[20] Lo Giudice G., Cutroneo G., Centofanti A. (2015). Dentin morphology of root canal surface: a quantitative evaluation based on a scanning electronic microscopy study. *BioMed Research International*.

[21] Vertucci F. J. (1978). Root canal morphology of mandibular premolars. *The Journal of the American Dental Association*.

[22] Hu S., Duan L., Wan Q., Wang J. (2019). Evaluation of needle movement effect on root canal irrigation using a computational fluid dynamics model. *BioMedical Engineering OnLine*.

[23] Shen Y., Gao Y., Qian W. (2010). Three-dimensional numeric simulation of root canal irrigant flow with different irrigation needles. *Journal of Endodontics*.

[24] Hsieh Y. D., Gau C. H., Kung Wu S. F., Shen E. C., Hsu P. W., Fu E. (2007). Dynamic recording of irrigating fluid distribution in root canals using thermal image analysis. *International Endodontic Journal*.

[25] Dioguardi M., Gioia G. D., Illuzzi G., Laneve E., Cocco A., Troiano G. (2018). Endodontic irrigants: different methods to improve efficacy and related problems. *European Journal of Dentistry*.

[26] Tay F. R., Gu L.-S., Schoeffel G. J. (2010). Effect of vapor lock on root canal debridement by using a side-vented needle for positive-pressure irrigant delivery. *Journal of Endodontics*.

[27] Dioguardi M., Di Gioia G., Illuzzi G. (2019). Passive ultrasonic irrigation efficacy in the vapor lock removal: systematic review and meta-analysis. *Scientific World Journal*.

[28] Srikanth P., Krishna A. G., Srinivas S., Reddy E. S., Battu S., Aravelli S. (2015). Minimal apical enlargement for penetration of irrigants to the apical third of root canal system: a scanning electron microscope study. *Journal of International Oral Health: JIOH*.

[29] Boutsioukis C., Gogos C., Verhaagen B., Versluis M., Kastrinakis E., Van Der Sluis L. W. M. (2010). The effect of apical preparation size on irrigant flow in root canals evaluated using an unsteady computational fluid dynamics model. *International Endodontic Journal*.

[30] Brunson M., Heilborn C., Johnson D. J., Cohenca N. (2010). Effect of apical preparation size and preparation taper on irrigant volume delivered by using negative pressure irrigation system. *Journal of Endodontics*.

[31] Zeng C., Willison J., Meghil M. M. (2018). Antibacterial efficacy of an endodontic sonic-powered irrigation system: an in vitro study. *Journal of Dentistry*.

[32] Chen J. E., Nurbakhsh B., Layton G., Bussmann M., Kishen A. (2014). Irrigation dynamics associated with positive pressure, apical negative pressure and passive ultrasonic irrigations: a computational fluid dynamics analysis. *Australian Endodontic Journal*.

[33] Van Der Sluis L. W. M., Versluis M., Wu M. K., Wesselink P. R. (2007). Passive ultrasonic irrigation of the root canal: a review of the literature. *International Endodontic Journal*.

